# Dynamic changes of periostin and collagen I in the sclera during progressive myopia in guinea pigs

**DOI:** 10.5935/0004-2749.20200034

**Published:** 2020

**Authors:** Bo Jiang, Chun-Sheng Shi

**Affiliations:** 1 Department of Ophthalmolohy, Anhui NO. 2 Provincial People’s Hospital, Hefei 230041, Anhui Province, China

**Keywords:** Myopia, Sclera, Guinea pigs, Periostin, Collagen type I, Miopia, Esclera, Cobaias, Periostina, Colágeno tipo I

## Abstract

**Purpose:**

To investigate periostin and collagen I expression during a scleral remodeling in myopic eyes and to determine their role in collagen remodeling of the myopic sclera.

**Methods:**

Fifty one-week-old guinea pigs were divided into the control and form-deprivation myopia (FDM) groups. The eyes of animals in the form-deprivation myopia group were covered for 2, 4, and 8 weeks, or were covered for 4 weeks and then uncovered for 2 weeks. The diopters and axial lengths in the eyes in each group of guinea pigs were measured. Immunohistochemistry and reverse transcription polymerase chain reaction were used to detect the relative protein and mRNA expressions of periostin and collagen I in the scleral tissues of guinea pig.

**Results:**

Before masking, guinea pigs in the control and form-deprivation myopia groups were hypermetropic and did not differ significantly (p>0.05). Hypermetropic refraction in the control group gradually decreased. In guinea pigs from the form-deprivation myopia group, the refractive power gradually changed from +2.14 ± 0.33 D to -7.22 ± 0.51 D, and the axial length gradually changed from 5.92 ± 0.37 mm to 8.05 ± 0.34 mm from before until the end of masking. Before covering, no significant difference was observed in the relative collagen I and periostin mRNA and protein expression levels in the sclera of the guinea pig control and form-deprivation myopia groups (p>0.05). The relative collagen I and periostin protein and mRNA expression levels in the sclera of guinea pigs in the form-deprivation myopia group at 2, 4, and 8 weeks, and after covering the eyes for 4 weeks followed by uncovering for 2 weeks, were significantly lower than those in the control group (p<0.05). The collagen I and periostin mRNA expression levels were positively correlated with protein expression levels in the sclera of guinea pigs (protein: r=0.936, p<0.05; mRNA: r=0.909, p<0.05).

**Conclusions:**

Periostin was expressed in the myopic sclera of guinea pigs, and changes in periostin and collagen I expression were highly consistent. Periostin and collagen I may be involved in the regulation of scleral remodeling in myopia.

## Abstract (pt)

**Objetivo:**

Investigar a expressão da periostina e do colágeno I durante o remodelamento escleral em olhos míopes e determinar seu papel na remodelação do colágeno da esclera miópica.

**Métodos:**

Cinquenta cobaias com uma semana de idade foram divididas em grupo controle e miopia de privação de forma. Os olhos dos animais no grupo de miopia de privação de forma foram cobertos por 2, 4 e 8 semanas, ou foram cobertos por 4 semanas e depois descobertas por 2 semanas. As dioptrias e comprimentos axiais dos olhos em cada grupo de cobaias foram medidos. A imunohistoquímica e a reação em cadeia da polimerase com transcrição reversa foram utilizadas para detectar as expressões relativas de proteína e mRNA de periostina e colágeno I em tecidos esclerais das cobaias.

**Resultados:**

Antes do mascaramento, as cobaias nos grupos controle e miopia de privação de forma eram hipermetrópicas e não diferiam significativamente (p>0,05). A refração hipermetrópica no grupo controle diminuiu gradualmente. Nas cobaias do grupo de miopia de privação de forma, a potência de refração mudou gradualmente de +2,14 ± 0,33 D para -7,22 ± 0,51 D e o comprimento axial mudou gradualmente de 5,92 ± 0,37 mm para 8,05 ± 0,34 mm desde antes até o final do mascaramento. Antes do mascaramento, nenhuma diferença significativa foi observada nos níveis de expressão de mRNA e proteína de colágeno I e periostina na esclera dos grupos controle e miopia de privação de forma (p>0,05). Os níveis relativos de expressão de colágeno I e proteína periostina e mRNA na esclera de cobaias no grupo de miopia de privação de forma em 2, 4 e 8 semanas, e após cobertura dos olhos por 4 semanas seguido de descoberta por 2 semanas, foram significativamente menores que aqueles no grupo controle (p<0,05). Os níveis de expressão de mRNA, colágeno I e proteína periostina foram positivamente correlacionados com os níveis de expressão de proteína na esclera das cobaias (proteína: r=0,936, p<0,05; mRNA: r=0,909, p<0,05).

**Conclusões:**

A periostina foi expressa na esclerótica míope de cobaias e as alterações na expressão de periostina e colágeno I foram altamente consistentes. A periostina e o colágeno I podem estar envolvidos na regulação do remodelamento escleral na miopia.

## INTRODUCTION

Myopia has the highest incidence among the global refractive diseases, especially in Asia and Southeast Asia, which have the highest incidence globally, comprising 20% of patients with high myopia^(1-2)^. By 2050, 4.785 billion people (49.8% of the world’s population) is estimated to suffer from myopia and 938 million people (9.8% of the world’s population) will suffer from high myopia^(3)^. Therefore, the etiological study on myopia that explored its pathogenesis, prevention, and treatment should be urgently addressed by ophthalmologists.

Nowadays, myopia occurred as a result of combined genetic and environmental factors. Axial elongation caused by scleral remodeling in myopic eyes is the main pathological mechanism for the progression of high myopia^(4-6)^. With advances in studies on molecular biology, several bioactive molecules have been found to play an important role in remodeling the posterior scleral pole in myopia. Studies have confirmed that three subtypes of transforming growth factor-β (TGF-β1, TGF-β2, and TGF-β3) all play an important role in scleral remodeling. Among them, TGF-β1 is the current research focus^(7-8)^. PN is a signal transduction gene downstream of TGF-β1 that plays a role in the synthesis and degradation of collagen I; however, details of its interaction with TGF-β1 during the myopia progression remain unclear^(9-10)^.

Therefore, this study established a form-deprivation myopia (FDM) model in guinea pigs to investigate the PN expression in the sclera of myopic eyes and its role in the synthesis and degradation of collagen I. Dynamic changes of PN expression in the sclera in relation to collagen I gene expression were investigated at different periods when the FDM was established in guinea pigs to explore the possible role and mechanism of PN in myopia progression.

## METHODS

### Animals and controls

Fifty healthy 1-week-old guinea pigs with body weights of 100-140 g were selected, which were randomly divided into five groups comprising 10 each. Those in the FDM group were covered with translucent latex balloons, in which holes were cut to expose the right eye, nose, lips, and ears. The left eyes remained covered for 2, 4, and 8 weeks, or were uncovered for 2 weeks after being covered for 4 weeks to prepare a monocular FDM model. Balloons were capped on the neck using a stapler and folded to prevent them from slipping or rotating. The FDM group was composed of guinea pigs with covered eyes, whereas those with uncovered eyes on the contralateral side consisted the control group. Animals were raised at the Experimental Animal Center of Anhui No. 2 Provincial People^’^s Hospital. The housing environment provided a 12-h natural lighting/12-h dark environment, with days starting at 8:00 am during the experiment. Water, vegetables, and vitamins were provided ad libitum. The room temperature was maintained at 22°C. Experimental procedures were in accordance with the “Guide for the Care and Use of Laboratory Animals” (published in May 2016). This study was approved by the Experimental Animal Ethics Committee of Anhui No. 2 Provincial People’s Hospital.

### Diopter and axial length measurements

Procedures were performed as previously described^(11-12)^. Measurements were taken from 10 guinea pigs from different groups at different times (before covering; after covering for 2, 4, and 8 weeks; and following uncovering for 2 weeks after 4 weeks of coverage). Tropicamide eye drops were used to fully dilate the pupils, and then streak retinoscopy (YZ24, Suzhou Liuliu Vision Technology Co., Ltd., China) was performed in the dark room. Diopters were accurate to 0.01 D. A-type ultrasound (AL-100, Japan TOMEY company, Japan) was used to measure the binocular axial length after the anesthesia administration (i.e., the distance from the corneal apex to the vitreoretinal interface in the posterior pole of the eyeball) in the manual mode. Measurements were obtained three times in a row, and the average value was rounded to the nearest 0.01 mm. All data were measured and recorded by experienced personnel.

### Immunohistochemistry study

Guinea pigs were sacrificed after anesthesia with 1% sodium pentobarbital; then, the eyes were removed, the anterior segment discarded, and the samples were placed on ice. The posterior sclera was excised around the head of the optic nerve using a 6-mm-diameter trephine, and the head of the optic nerve was discarded. Then, the scleral tissues were fixed in 40% formaldehyde solution at 4ºC. Samples were cut into sections, blocked, incubated with primary antibodies against PN and collagen I, and analyzed under a fluorescent microscope. The negative control used phosphate-buffered saline instead of the primary antibody. The evaluators followed the double-blind principle. An anti-rat PN antibody (1:150) (AD082529, Beijing Boaosen Biotechnology Co., Ltd.); Anti-rat collagen I antibody (1:150) (AA56131, Shanghai Baili Biotechnology Co., Ltd.); and General-purpose secondary antibody reagent Box (PV-6000) (K155922D, Beijing Zhongshan Golden Bridge Biotechnology Co., Ltd.) were used in the experiment.

### Reverse transcription PCR study

The guinea pigs in each group were sacrificed, by performing tissue acquisition as described above. At each time point, five eyes from each group were sampled, and the required amount of scleral tissue was ground in liquid nitrogen and used to extract the total RNA according to the QuantiFast SyBr Green PCR kit (151034942, Qiagen, Germany). The RNA was reverse transcribed by the RevertAidTM first Strand cDNA Synthesis Kit (00287813, Thermo, China), and the resultant cDNA was stored at -80°C until PCR analysis. Nucleotide sequences of the primers are shown in table 1. The PCR instrument (K960, Hangzhou Jingge Scientific Instrument Co., Ltd., China) was used to perform PCR amplification under the following cycling conditions: 95°C for 5 min, 35 cycles at 95°C for 30 s, and 72°C for 40 s. The amplified cDNA products were separated by agarose gel electrophoresis and their relative concentrations, estimated from fluorescence intensity, and analyzed using a gel imaging system. The experiment was repeated three times with the average values of mRNA recorded. β-actin was used as an internal control.

**Table 1:** Primer sequences and predicted product sizes

Gene	Primer (5’-3’)	Size (bp)
β-actin	F: GCTCTATCCTGGCCTCACTC R: GGGTGAGGGACTTCCTGTAA	400
collagen I	F: ACAAGCGATTACACACCCAA R: TTAGTTTCCTGCCTCTGCCT	239
PN	F: CCACTGCCAGTTCTCTTCGT R: GGACTTAACTGTAGCGGAGA	486

### Statistical analysis

The SPSS 22.0 statistical software was used for statistical analysis. Measurement data in this study were tested for normality using the Shapiro-Wilk test and expressed as the mean ± SD. The paired t-test was used to analyze differences. The Pearson linear correlation analysis was used to evaluate the relationship between PN and collagen I and PN mRNA expression in the scleral tissue of guinea pigs in the FDM group, with hypothesis tests performed on correlation coefficients. The double-guard assay was used, with P<0.05 considered statistically significant.

## RESULTS

### Diopter and axial length

When guinea pigs were born, hyperopia was initiated in their eyes, with comparatively short axial lengths. By inducing FDM, the refractive status in the FDM group gradually changed from hyperopia to myopia (p<0.05, t-test). However, the hyperopic refraction of guinea pigs in the control group gradually decreased. The difference in refractive power and axial length between the FDM and control groups before the eye masking was not significant (p>0.05). However, differences in refractive power and axial length between the FDM and control groups were significant at 2, 4, and 8 weeks, and 2 weeks after a 4-week masking treatment (p<0.05, Table 2).

**Table 2:** Refractive state and axial length between the eyes of the two groups (± s)

Group	Refractive power (D, n=10)		t	p-value	Axial length (mm, n = 10)		t	p-value
	Control	FDM			Control	FDM		
0 weeks	2.15 ± 0.31	2.14 ± 0.33	0.26	0.79	5.91 ± 0.35	5.92 ± 0.37	-0.09	0.93
2 weeks	1.31 ± 0.33	-1.27 ± 0.69	12.49	0.00	6.27 ± 0.36	6.65 ± 0.34	-2.59	0.03
4 weeks	0.46 ± 0.23	-4.19 ± 0.60	28.33	0.00	6.42 ± 0.32	7.24 ± 0.27	-6.72	0.00
2 after 4 weeks	0.11 ± 0.27	-4.64 ± 0.61	28.47	0.00	6.54 ± 0.29	7.31 ± 0.31	-6.18	0.00
8 weeks	-0.39 ± 0.25	-7.22 ± 0.51	45.63	0.00	7.07 ± 0.31	8.05 ± 0.34	-6.74	0.00

### Protein expression of PN and collagen I

**Figure 1:**
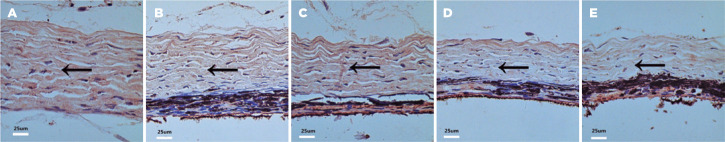
Expression and distribution of collagen I in the posterior sclera of guinea pigs. (A) Collagen I expression in the sclera of the posterior pole was observed in the control group, as shown in brown staining (arrow) (B) Collagen I expression in the sclera of the posterior pole was lower in the FDM group than that in the control group after 2 weeks (arrow) (C) After 4 weeks of masking, the collagen I expression in the posterior sclera in the FDM group was reduced (arrow) (D) After being uncovered for 2 weeks after being masked for 4 weeks, collagen I expression in the posterior sclera of guinea pigs from the FDM group decreased (arrow) (E) After 8 weeks of masking, collagen I expression in the posterior sclera of the FDM group was reduced (arrow). DAB ×200, scale bar= 25 µm.

**Figure 2:**
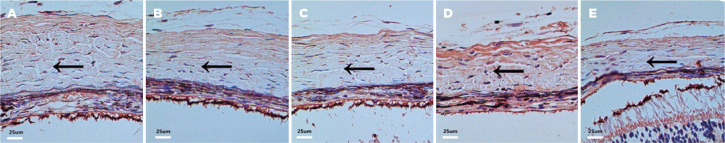
Expression and distribution of PN in the posterior sclera of guinea pigs in each group. (A) The PN expression in the sclera of the posterior pole was observed in the control group, showing brown staining (arrow) (B) In the FDM group, the PN expression in the sclera of the posterior pole was lower than that in the control group at 2 weeks (arrow) (C) After 4 weeks of masking, the PN expression in the posterior sclera of the FDM group was reduced (arrow) (D) After being uncovered for 2 weeks after 4 weeks of masking, the PN expression in the posterior sclera of the guinea pigs from the FDM group decreased (arrow) (E) After 8 weeks of masking, the PN expression in the posterior sclera of the FDM group was reduced (arrow). DAB ×200, scale bar = 25 µm.

The collagen I and PN protein expression of in the scleral fibrosis tissue of guinea pigs in the FDM and control groups is indicated by brown staining (Figure 1 and 2). As the FDM time increases, the expression intensity of collagen I and PN protein in scleral tissues decreased (Figure 1 and 2). Before covering the eyes, no significant difference was observed in the collagen I and PN protein expression between the FDM and control groups (p>0.05). After being covered for 2, 4, and 8 weeks, and uncovered for 2 weeks after 4 weeks of masking, the collagen I and PN protein expression levels in the FDM group were significantly lower than those in the control group. These differences were statistically significant (p<0.05, Table 3).

**Table 3:** Comparison of protein expression of collagen I and PN in the eyes of the two groups (± s)

Group	Collagen I (n=10)		t	p-value	PN (n = 10)		t	p-value
	Control	FDM			Control	FDM		
0 weeks	0.907 ± 0.041	0.925 ± 0.025	-2.00	0.12	0.680 ± 0.065	0.793 ± 0.079	-1.79	0.15
2 weeks	0.884 ± 0.019	0.750 ± 0.024	7.45	0.00	0.642 ± 0.060	0.542 ± 0.026	3.59	0.02
4 weeks	0.858 ± 0.025	0.551 ± 0.025	19.47	0.00	0.631 ± 0.048	0.370 ± 0.02	9.21	0.00
2 after 4 weeks	0.850 ± 0.022	0.478 ± 0.040	13.21	0.00	0.605 ± 0.056	0.369 ± 0.019	7.12	0.00
8 weeks	0.837 ± 0.016	0.410 ± 0.043	16.97	0.00	0.581 ± 0.061	0.336 ± 0.047	5.37	0.01

Note: (paired t-test) FDM= form-deprivation myopia.

### PN and collagen I mRNA expression

**Figure 3:**
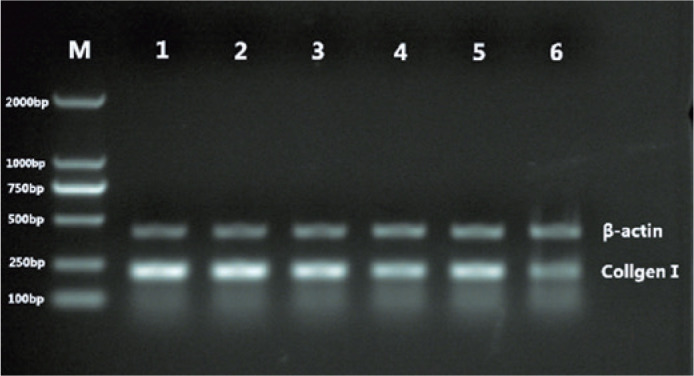
Changes in collagen I mRNA expression in the sclera of guinea pigs in different groups.

**Figure 4:**
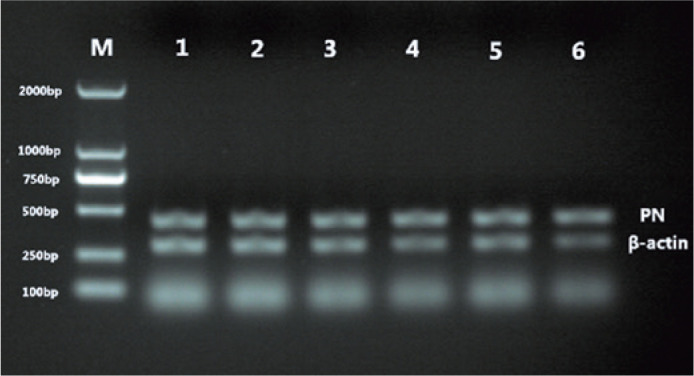
Changes in PN mRNA expression in the sclera of guinea pigs in different groups.

Collagen I and PN mRNA were expressed in the posterior pole sclera of guinea pigs from both the FDM and control groups. In the FDM group, the expression gradually decreased as the modeling time increased (Figures 3 and 4). Before covering the guinea pigs’ eyes, the difference in collagen I and PN mRNA expression was not significant between the FDM and control groups (p>0.05). After masking for 2, 4, and 8 weeks, and uncovering for 2 weeks after 4 weeks of masking, they were significantly lower in the FDM group than those in the control group (p<0.05, Table 4).

**Table 4:** mRNA expression of collagen I and PN between the eyes of the two groups (± s)

Group	Collagen 1 (n = 10)		t	i-value	PN (n=10)		t	p-value
	Control	FDM			Control	FDM		
0 weeks	1.320 ± 0.012	1.337 ± 0.017	-0.32	0.76	0.960 ± 0.022	0.973 ± 0.013	-0.95	0.40
2 weeks	1.265 ± 0.058	0.931 ± 0.011	11.32	0.00	0.934 ± 0.011	0.792 ± 0.020	26.69	0.00
4 weeks	1.220 ± 0.042	0.888 ± 0.027	24.18	0.00	0.902 ± 0.008	0.567 ± 0.024	39.59	0.00
2 after 4 weeks	1.203 ± 0.056	0.859 ± 0.045	33.41	0.00	0.883 ± 0.022	0.526 ± 0.022	26.75	0.00
8 weeks	1.191 ± 0.059	0.743 ± 0.043	27.26	0.00	0.863 ± 0.017	0.467 ± 0.022	58.27	0.00

Note: (paired t-test) FDM: form-deprivation myopia.

M= Molecular weight size marker; 1= right eye at 0 weeks; 2= left eye at 0 weeks; 3= 2 weeks; 4= 4 weeks; 5= 2 weeks after 4 weeks of masking; and 6= 8 weeks.

M= Molecular weight size marker: 1= right eye at 0 weeks; 2= left eye at 0 weeks; 3= 2 weeks; 4= 4 weeks; 5, 2 weeks after 4 weeks of masking; 6= 8 weeks.

### Correlation analysis of Sp1 and collagen I expression in FDM

In the scleral tissue, collagen I and PN protein expressions were significantly correlated (r=0.936, p<0.05), and collagen I and PN mRNA expressions were also significantly correlated (r=0.909, p<0.05).

## DISCUSSION

Previous studies have shown that scleral remodeling plays a crucial role in the occurrence and progression of myopia. Axial elongation is the most important form of changes in the progression of myopia^(13-14)^. Collagen fibers are the most abundant constituents in the sclera, accounting for 90% of its net weight. These fibers are primarily formed by collagens Ι, III, and IV, with collagen Ι fibers occupying the largest area of the sclera. In myopic scleral remodeling, collagen expression, especially that of collagen Ι, decreases^(15-17)^. Studies have confirmed that TGF-β plays an important role in maintaining the normal morphology and function of the sclera^(18)^. TGF-β1, TGF-β2, and TGF-β3 expressions in the sclera all decreased in the occurrence of myopia^(19-20)^.

FDM was induced at different times by masking with translucent latex balloons that covered the animal head and the left eye, whereas the right eye, nose, mouth, and ears were exposed. The results in this study showed that, the degree of myopia in the guinea pigs was significantly higher and the axial length was significantly elongated in the FDM than those in the control group, which confirmed the results in our previous study^(12)^. With the prolongation of the masking time, the degree of myopia refraction in guinea pigs in the FDM group gradually increased, accompanied by gradual extension of the axial length, confirming that FDM was primarily formed via an abnormal increase in the axial length.

Studies have shown that in the process of scleral remodeling in myopia, TGF-β1 and collagen I expressions gradually decrease as the degree of myopia increases, with a certain degree of correlation between them^(21)^. The results in this study show that protein and mRNA expression of PN and collagen I gradually decreased in the posterior pole of guinea pigs as masking time increased. This shows that PN and collagen I expressions in the sclera gradually decrease as the degree of myopia increases. However, 1 week after the 4-week treatment, PN and collagen I expression levels were similar to the levels observed at 4 weeks, indicating that the reduction rate had slowed down. Masking is suspected to increase the degree of myopia and axial length in guinea pigs, leading to a gradual decrease in PN and collagen I expressions in the sclera.

In conclusion, our study confirms that PN is expressed in guinea pigs’ sclera with experimental myopia, and PN protein and gene expression in the scleral tissues of FDM gradually decreased with prolongation of the masking time and deepening of the degree of myopia. The correlation between PN and type I collagen expression suggests that PN is involved in a pathway that regulates type I collagen synthesis in myopic scleral remodeling. It can be speculated that the TGF-β1-PN signaling pathway may be involved in scleral remodeling and myopia pathogenesis. In the future, we will conduct an in-depth study on TGF-β1 changes in the guinea pigs with myopia sclera and how the TGF-β1-PN pathway regulates type I collagen synthesis. We recommend that future studies use a larger sample size to further validate these results.
